# Comparison of Multiple Methods for Determination of *FCGR3A/B* Genomic Copy Numbers in HapMap Asian Populations with Two Public Databases

**DOI:** 10.3389/fgene.2016.00220

**Published:** 2016-12-26

**Authors:** Yuan-yuan Qi, Xu-jie Zhou, Ding-fang Bu, Ping Hou, Ji-cheng Lv, Hong Zhang

**Affiliations:** ^1^Renal Division, Peking University First HospitalBeijing, China; ^2^Peking University Institute of NephrologyBeijing, China; ^3^Key Laboratory of Renal Disease, Ministry of Health of ChinaBeijing, China; ^4^Key Laboratory of Chronic Kidney Disease Prevention and Treatment, Peking University, Ministry of EducationBeijing, China; ^5^Research Central Institute, Peking University First HospitalBeijing, China

**Keywords:** copy number, *FCGR3*, TaqMan qPCR assay, HapMap CHB, PRT

## Abstract

Low *FCGR3* copy numbers (CNs) has been associated with susceptibility to several systemic autoimmune diseases. However, inconsistent associations were reported and errors caused by shaky methods were suggested to be the major causes. In large scale case control association studies, robust copy number determination method is thus warranted, which was the main focus of the current study. In the present study, *FCGR3* CNs of 90 HapMap Asians were firstly checked using four assays including paralog ratio test combined with restriction enzyme digest variant ratio (PRT-REDVR), real-time quantitative (qPCR) using TaqMan assay, real-time qPCR using SYBR Green dye and short tenden repeat (STR). To improve the comparison precision reproductively, the results were compared with those from recently released sequencing data from 1000 genomes project as well as whole-genome tiling BAC array data. The tendencies of inconsistent samples by these methods were also characterized. Refined in-home TaqMan qPCR assay showed the highest correlation with array-CGH results (*r* = 0.726, *p* < 0.001) and the highest concordant rate with 1000 genome sequencing data (*FCGR3A* 91.76%, *FCGR3B* 85.88%, and *FCGR3* 81.18%). For samples with copy number variations, comprehensive analysis of multiple methods was required in order to improve detection accuracy. All these method were prone to detect copy number to be higher than that from direct sequencing. All the four PCR based CN determination methods (qPCR using TaqMan probes or SYBR Green, PRT, STR) were prone to higher estimation errors and thus may lead to artificial associations in large-scale case-control association studies. But different to previous reports, we observed that properly refined TaqMan qPCR assay was not inferior to or even more accurate than PRT when using sequencing data as the reference.

## Introduction

Fcγ receptors were cellular receptors, which were encoded by *FCGR3* locus, for the Fc region of IgG and IgE, and could transmit their signal by tyrosine-based activation (ITAM) or inhibitory motifs (ITIM). Low-affinity activating receptor FcγRIII genes, which encoded FcγRIIIA and FcγRIIIB, were located in 1q23.3 with extensive copy number variation (CNV) (Redon et al., 2006; Nimmerjahn and Ravetch, 2008). Low copy number of *FCGR3B* had been identified to be associated with a number of systemic autoimmune diseases, such as systemic lupus erythematosus (SLE), rheumatoid arthritis (RA), ANCA-associated vasculitis (AAV), and anti-glomerular basement (anti-GBM disease) disease (Fanciulli et al., 2007; Willcocks et al., 2008; Mamtani et al., 2010; McKinney et al., 2010; Niederer et al., 2010; Zhou et al., 2010, 2011; Molokhia et al., 2011; Chen et al., 2014). However, results for disease associations were not consistent, even for the same phenotype in populations within the same ancestry. Phenotype and genetic heterogeneity might be confounding factors. However, methodology discrepancies were suggested to be the major factor leading to the conflicting results. Certain proportion of errors might lead to false associations, especially when the effect of gene risk was moderate. Thus, in large-scale genetic association studies, easy, economic, robust, and accurate methods of *FCGR* CN genotyping were prerequisites.

Up to date, multiple assays for *FCGR* CN genotyping had been developed, including real-time quantitative PCR (qPCR), paralog ratio test combined with restriction enzyme digest variant ratio (PRT-REDVR), multiples ligation-dependent probe amplification (MLPA), short tenden repeat (STR), comparative genomic hybridization (CGH), and direct sequencing (Aitman et al., 2006; Fanciulli et al., 2007; Breunis et al., 2008, 2009; Willcocks et al., 2008; Mamtani et al., 2010). But due to high similarity and CN complexity in the region, no single method or single test was suggested to be ideal in fastness, accuracy and economy. Though sequencing data was the gold standard, it was not suitable for large scale case-control study considering the expenses. Our aim was to evaluate the efficacy of the widely used methods and find a robust assay with a low error rate for further large-scale case control genetic association study. In the current study, we determined the copy numbers for 90 HapMap Chinese Han from Beijing (CHB) and Japanese from Tokyo (JPT) individuals using four copy number assays: PRT-REDVR, TaqMan qPCR, SYBR Green qPCR, and STR (Fanciulli et al., 2007; Hollox et al., 2009; Zhou et al., 2010), as these methods were more widely taken and economical for large-scale association. We compared our results of each method with the copy numbers from established open database including the whole-genome tiling BAC array data and the 1000 genome sequencing data.

Notably, thanks to the 1000 Genomes Project phase 3, an integrated map of structural variation in 2504 human genomes which was constructed in 2015. It presented the most comprehensive set of human structural variants to date. As an integrated resource for disease and population genetic studies, it represented an invaluable resource for the construction and analysis of personalized genomes. In terms of method comparison, it was also of special importance, as it revealed CN variations at individual instead of pooling level (Sudmant et al., 2015). Thus, to improve the comparison efficiency, the sequencing data from 1000G samples represented as the gold standard available compared to previous reports replying on CGH data. In this way, we were aiming to find a method in *FCGR3* copy number detection which was the fastest and cheapest without scarifying accuracy.

## Materials and methods

### Subjects

The study population was based on the HapMap Phase II Han Chinese individuals from Beijing (CHB, *n* = 45) and Japanese individuals from Tokyo (JPT, *n* = 45). Genomic DNA was purchased from the Coriell Cell Repositories (Catalog ID: HAPMAPPT02). This study was approved by the Medical Ethics Committee of Peking University First Hospital.

### Real-time quantitative PCR (qPCR)

#### TaqMan qPCR system

*FCGR3B* and *FCGR3B* copy number (CN), determined by Quantitative PCR (qPCR), was performed on an Applied Biosystems 7500 (Foster City, CA, USA) as previously reported (Zhou et al., 2010). Primers and TaqMan probes specifically amplify the target gene were designed to avoid paralogous or allelic sequence variants. Coagulation factor V gene (F5) was included as an internal control for copy number. The qPCRs was in 50 ul reaction system including 80 ng genomic DNA, 5ul 10 × PCR buffer, 4 ul 2.5 mM deoxyribonucleoside triphosphate, 15 pmol/each forward primers, 15 pmol/each reverse primers, 5 pmol/each TaqMan probes, 1.5 U Taq polymerase (Takara, Dalian, China), and 26.7ul ddH_2_O. Cycling conditions were 94°C for 10 min, 40 cycles with 10 s denaturation step at 94°C, followed by 62°C (*FCGR3B* or *FCGR3B*) annealing step for 45 s and a 72°C extension step for 10 s (Zhou et al., 2010). The target gene and the control gene were amplified in the same tube and each test was run in triplicate. The standard curves to test the efficiency of the assay were run using independent genomic DNA (Zhou et al., 2010).

### SYBR Green qPCR system

*FCGR3B* copy number assay was also carried out by SYBR Green qPCR system, performed on an Applied Biosystems 7500 (Foster City, CA, USA) and analyzed by the relative standard curve method. The primers, qPCR reactions, and calculation were referred to previous reports (Aitman et al., 2006).

### Paralog ratio test (PRT)

The primer pair *FCGR3A*/*FCGR3B* co-amplified in *FCGR3A* and *FCGR3B*. The other primer pair, *FCGR3*/c18, amplified a sequence of the same length in both target genes and a third region in Chromosome 18 (Niederer et al., 2010). The amplification of 5–10 ng of genomic DNA in PCR reaction was performed using Phusion Hot Start High Fidelity polymerase (Finnzymes) with the HF buffer. The cycling conditions were 98°C for 60 s, then 30 cycles at 98°C for 10 s, annealing at 65°C (for *FCGR3A*/*FCGR3B*) or 60°C (for *FCGR3*/c18) for 20 s and elongation at 72°C for 30 s (for *FCGR3A*/*FCGR3B*) or 5 s (for *FCGR3*/c18), followed by 72°C for 7 min to reduce single stranded DNA products (Niederer et al., 2010). The PCR products were added to Hi-DiTM Formamide with the fluorescent GeneScanTM 500 LIZTM Size Standard (Applied Biosystems) and the following analysis were performed on ABI 3730 × l DNA Analyzer/Sequencer (Applied Biosystems). Peak areas corresponding to the two products were recorded for HEX-labeled products using GeneMapper 4.0 software (Applied Biosystems).

### Restriction enzyme digest variant ratio (REDVR)

In the present study, we performed two REDVR assays in order to distinguish the variants from *FCGR3B* and *FCGR3B* (C733 Arginine > Stop) and to distinguish human neutrophil antigens (HNA) HNA1a and HNA1b on *FCGR3B* (C147T). The primer sequences were referred to previously published article (Hollox et al., 2009). We amplified the two regions in duplex using the same conditions as the PRT described above with some modification. Copy number calls were estimated based on mean ratios of the product and the reference standard for experimental calibration. A maximum likelihood approach was used to analyze the PRT copy number calls in combination with the REDVR analysis.

### Short tandem repeat (STR)

The primers for STR could be referred to previously report (Hollox et al., 2009). The PCR condition was based on PRT with some modifications, that was, 26 cycles for amplification and 2 μl for capillary electrophoresis analysis. During amplification, the wrong replication of Taq DNA polymerase would generate slippage peaks in MSAT1 amplification. The 2 bp larger real peak would add to the area under the slippage peak in case that the two peaks failed to overlap. It was difficult to distinguish a slippage peak from a real peak which were 2 bp. Given that the amount of slippage peak was dependent on the proportional to variant length, we corrected a length dependent factor to real peaks which coinciding with slippage peaks. If it showed more than two peaks, the peak which had the second smallest area was used to divide the area of each peak. If it showed more than four peaks, the peak with the third smallest area was used to divide the area of each peak. In this way, we determined the value for copy numbers after slippage correction.

## Results

### Respective comparisons among all the 4 PCR based CN determination assays

In the present study, we performed 4 methods with 3 repeats (TaqMan qPCR, PRT-REDVR, SYBR Green qPCR, and STR) to estimate the copy numbers for *FCGR3* locus in CHB (*n* = 45) and JPT (*n* = 45) populations (detailed copy number results for *FCGR3A, FCGR3B*, and *FCGR3* by each method was shown in Table S1).

We next analyzed the concordant rate for *FCGR3A, FCGR3B*, and *FCGR3* with two or three assays (TaqMan qPCR, PRT, REDVR, SYBR Green qPCR).

In two assay concordant analysis, TaqMan qPCR showed the highest concordant rate with other methods. The concordant rates were 77.78 ~ 91.11% (91.11% for *FCGR3A*, 84.44% for *FCGR3B*, and 77.78% for *FCGR3*) between TaqMan qPCR and PRT-REDVR, 74.44% for *FCGR3B* between TaqMan qPCR and SYBR Green, and 76.67% for *FCGR3* between TaqMan qPCR and STR. SYBR Green (74.44% with TaqMan qPCR and 65.56% with PRT-REDVR for *FCGR3B*) and STR (76.67% with TaqMan qPCR and 61.11% with PRT-REDVR for *FCGR3*) showed the lowest concordant rate with other methods. In three assay analysis, the concordant rates were much poorer, 62.22% for *FCGR3B* (TaqMan qPCR, PRT-REDVR and SYBR Green) and 60.00% for *FCGR3* (TaqMan qPCR, PRT-REDVR and STR). This result was consistent with previous observations (Haridan et al., 2015).

Not only TaqMan and SYBR Green were qPCR based methods, PRT, REDVR, and STR were also partly qPCR based in methodology. They might skew toward same mistakes in copy number detection. In general, even if these methods showed good concordant rate with each other, it was hard to determine which one was the most suitable method in *FCGR3A, FCGR3B*, and *FCGR3* copy number detection. In order to solve the problem, all the results by these methods (TaqMan qPCR, PRT-REDVR, and STR) should be tested with a “gold standard” in paired samples.

### Correlation with the whole-genome tiling BAC array data

**Figure 1:**
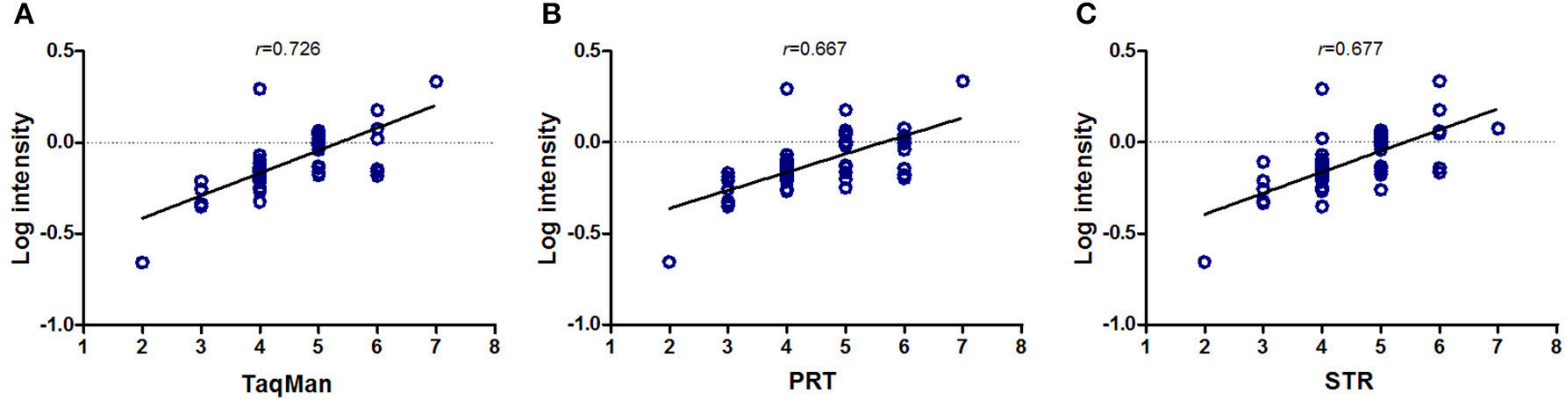
**Correlation analysis of copy number measurements with array-CGH**. TaqMan qPCR (**A**, *r* = 0.726), PRT-REDVR (**B**, *r* = 0.667) and STR (**C**, *r* = 0.677) were significant correlated with array-CGH results (*p* < 0.001).

We used whole-genome tiling BAC array data from the Wellcome Trust Sanger Institute in paired samples as reference, which was more widely used in previous method reports (Redon et al., 2006). As shown in Figure 1, all of TaqMan qPCR, PRT and STR results showed a significant correlation with array-CGH results (*p* < 0.001). Amongst, TaqMan qPCR assay showed the highest correlation with array-CGH results with *r* = 0.726 (*p* < 0.001). There was also a significant correlation between PRT (*r* = 0.667, *p* < 0.001), STR (*r* = 0.677, *p* < 0.001) with the array-CGH results (Figure 1). It also indicated that all the four methods had good performance for CN calculation in tendency.

### Correlation with the 1000 genome sequencing data

**Figure 2:**
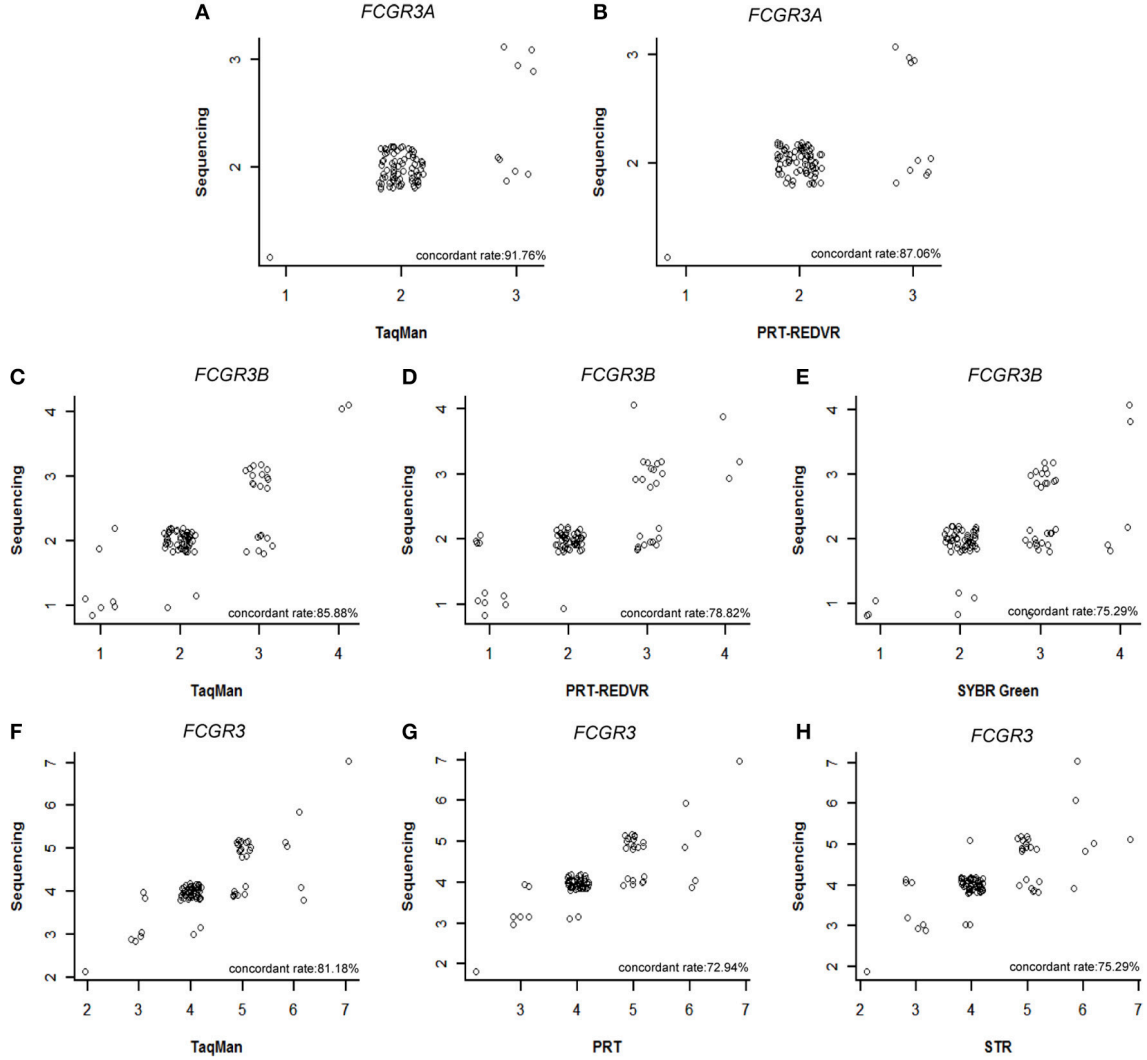
**Concordant analysis of CNV measurement with 1000 genome sequencing data**. Concordant rate between 1000 genome sequencing data and PRT-REDVR of *FCGR3A* **(A)**, 1000 genome sequencing data and TaqMan qPCR of *FCGR3A* **(B)**, 1000 genome sequencing data and PRT-REDVR of *FCGR3B* **(C)**, 1000 genome sequencing data and SYBR qPCR of *FCGR3B* **(D)**, 1000 genome sequencing data and TaqMan qPCR of *FCGR3B* **(E)**, 1000 genome sequencing data and PRT of *FCGR3* **(F)**, 1000 genome sequencing data and STR of *FCGR3* **(G)**, 1000 genome sequencing data and TaqMan qPCR of *FCGR3* **(H)**.

However, copy numbers were integers instead of linear ones. We conducted a concordant analysis between our TaqMan qPCR, PRT, REDVR, STR, SYBR Green qPCR results of *FCGR3A, FCGR3B, FCGR3* and the sequencing data from the 1000 genome phase-3-structural-variant-dataset in paired samples (Sudmant et al., 2015) (Figure 2). A significantly increased inconsistency was observed for every method.

For single method precision, TaqMan qPCR assay still showed the highest concordant rate with the sequencing results for *FCGR3A* (91.76%), *FCGR3B* (85.88%), and *FCGR3* (81.18%). PRT-REDVER showed the lowest concordant rate for *FCGR3* (72.94%). The concordant rate was also low in SYBR Green qPCR assay for *FCGR3B* (75.29%) and STR assay for *FCGR3* (75.29%).

Error was observed in almost every copy number detection assay (Figure 2). Although there was 0–5% error rate for wrong classification of varied copy numbers, it was strange that the highest error rate was observed in *FCGR3A* and *FCGR3B* 2-copy (8.24–20.00% error rate) and *FCGR3* 4-copy (14.12–17.65%). For 2-copy detection, TaqMan showed the lowest error rate 8.24% for *FCGR3A*, 11.76% for *FCGR3B*, and 14.12% for *FCGR3*. PRT showed a higher error rate in 2-copy detection, 12.94% for *FCGR3A*, 16.47% for *FCGR3B*, and 17.65% for *FCGR3*. The SYBR Green showed the highest error rate with 20.00% in *FCGR3B* 2-copy detection. This observation suggested that erroneous copy number determination might be increased due to wrong counting of the diploid nature of the genome.

**Figure 3:**
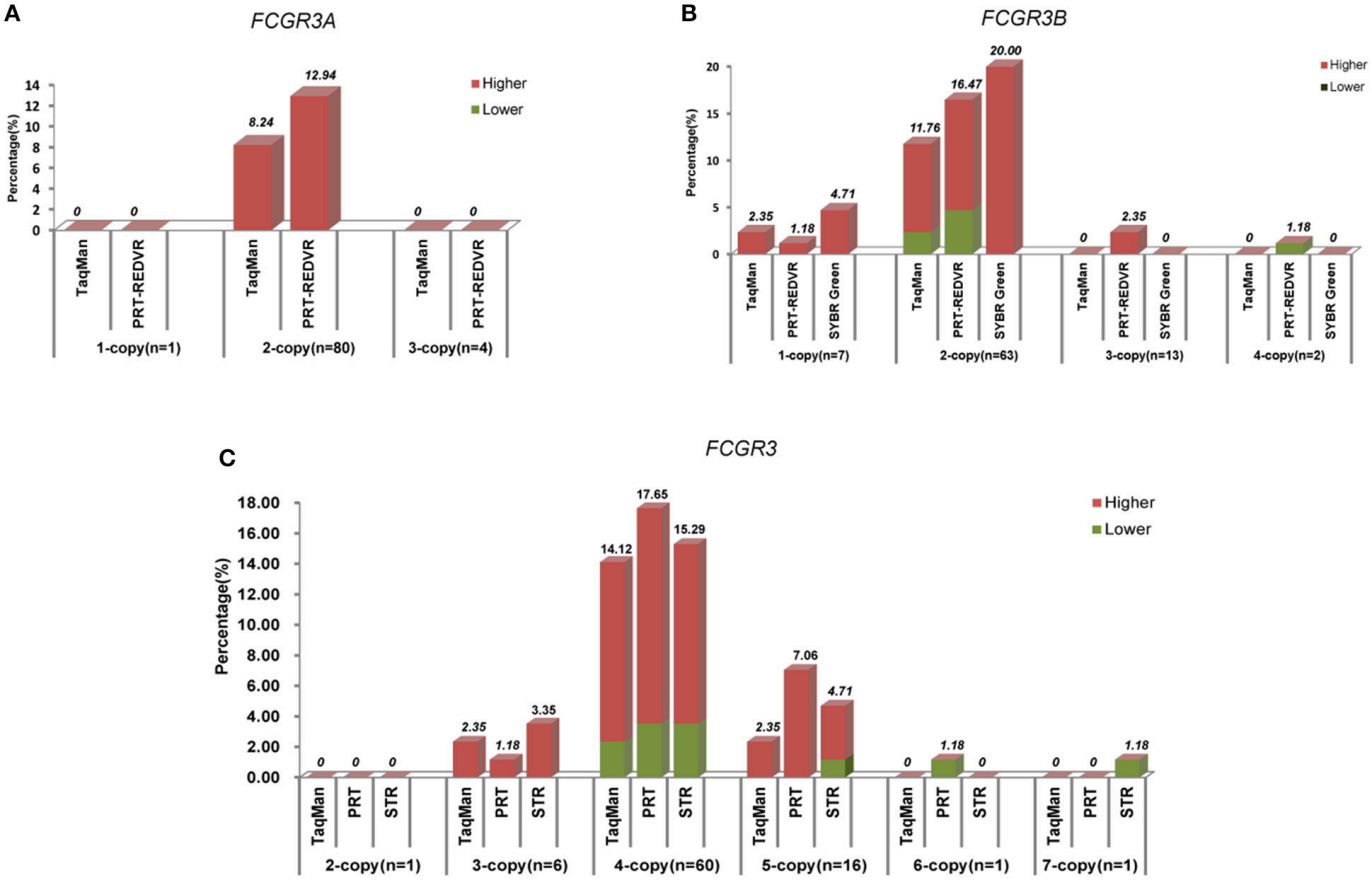
**Characteristics of wrong detected copy numbers**. The percentage (%) of wrong detected copy number by every measurement for each copy of *FCGR3A* **(A)**, *FCGR3B* **(B)**, and *FCGR3* **(C)** was labeled above the column. If the copy number were detected higher than the sequencing data, the column would be colored red. If the copy number were detected lower than the sequencing data, the column would be colored green.

And in all methods, the wrong detections tended to be higher estimation (Figure 3). 2-copy for *FCGR3A*, 1-copy and 2-copy for *FCGR3B*, and 3-copy, 4-copy, and 5-copy for *FCGR3* were skewing toward higher estimated in all the utilized methods. *FCGR3A* showed that wrong detected samples by TaqMan qPCR and PRT-REDVR were all estimated to be higher. For *FCGR3B* and *FCGR3* wrong detected samples, both higher and lower estimations were observed in TaqMan qPCR, PRT, REDVR, and STR, in which higher estimation took up the majority. 100% SYBR Green wrong detected samples were higher estimated. There was a tendency that samples with lower (2-copy for *FCGR3A*, 1-copy, 2-copy, 3-copy for *FCGR3B*, and 2-copy, 3-copy, 4-copy, and 5-copy for *FCGR3*) copy number were prone to higher estimation. Samples with higher copy numbers (4-copy for *FCGR3B*, 6-copy and 7-copy for *FCGR3*) were tended to be lower estimated.

## Discussion

In the present study, after the comparison of PRT-REDVR, TaqMan qPCR, SYBR Green qPCR, and STR, we found that TaqMan qPCR was the statistically supported method which could detect the copy number more accurately at *FCGR3* region in the HapMap CHB and JPT population in our institute. qPCR was widely used and criticized by its veracity and reliability as it could potentially introduce false positive calls. In our research, this problem might be involved in our SYBR Green qPCR assay. SYBR Green qPCR showed the lowest concordant rate and correlation with results from database in *FCGR3B* detection. Therefore, we refined the method of TaqMan qPCR in *FCGR3A, FCGR3B*, and *FCGR3* copy number determination. And TaqMan qPCR showed the highest concordant rate with 1000 genome sequencing data and correlation with the array-CGH results. Our data showed that TaqMan qPCR assay might be an option for future high throughput case-control study.

We used two standard populations from the HapMap project, 45 CHB and 45 JPT individuals. Previous studies by other groups also defined the copy number of HapMap CHB and JPT populations. We also compared the previously reported results with 1000 genome sequencing data and array-CGH result in paired samples (Hollox et al., 2009). The correlation between previously reported data and array-CGH results was 0.618 (*p* < 0.001) and the concordant rate was 85.88% for *FCGR3A*, 78.82% for *FCGR3B* and 70.59% for *FCGR3* comparing with 1000 genome sequencing data. The differences in copy number detection in one sample by the four methods (Taqman, SYBR Green, PRT-REDVR, and STR) individually could reflect either measurement error or real copy number heterogeneity between repeats. Meanwhile, the integration of multiple methods could improve the accuracy. The integrated copy number *FCGR3* showed 89.4% consistency with the 1000 genome sequencing data which was higher than using PRT (72.94%), STR (75.29%), and Taqman (81.18%) alone. The integrated copy number *FCGR3* also showed higher (0.932) correlation with the whole-genome tiling BAC array data than using PRT (0.667), STR (0.677), and Taqman (0.726) alone. Comparing with the result of single method, the integration by several methods would surely improve the accuracy. Our PRT and STR results were successfully replicated the previous reported data which also based on these two methods. However, TaqMan qPCR refined by our laboratory showed better performance than PRT. Our finding suggested that before case-control association studies, methodologies should be confirmed with standard population. That is to detect certain gene copy number using multiple methods in the standard population and followed by comparing the results with array-CGH results or 1000 genome sequencing data for methodology evaluation. Array-CGH reflects copy numbers based on relative dosage signal and the 1000 genome sequencing data precisely presented every copy number for each sample from a certain locus. Array-CGH had been utilized for detection evaluation by other groups (Hollox et al., 2009). However, we proposed that 1000 genome sequencing data would be a better option and array-CGH could be an implement in methodology evaluation. In this way, any laboratory could find a suitable method for copy number detection with minimum error rate. And method refinement would further improve the accuracy of detection.

Our study aims to find a better way to determine *FCGR3* genomic copy number in Asia populations. Nevertheless, TaqMan qPCR, PRT-REDVR, STR, SYBR Green qPCR were prone to detect a higher copy number. Therefore, in order to improve the accuracy in copy number determination in samples with high copy numbers, a combination of two or more methods was required. Our refined TaqMan qPCR alone was statistically supported as an option in *FCGR3* copy number determination for future case-control association studies. Advantages include small amount of DNA, high-throughput analysis, low running cost, and feasibility of equipment. However, this method could potentially introduce false positive results and DNA integrity should also be warranted. We hoped such endeavor will benefit future case-control studies with regard to complex CNVs, particularly for disease associations with genetic variations of FcγRs.

## Author contributions

XZ and HZ designed the research; XZ, DB, PH, JL and YQ performed the research; YQ analyzed the data; and YQ and XZ wrote the paper.

### Conflict of interest statement

The authors declare that the research was conducted in the absence of any commercial or financial relationships that could be construed as a potential conflict of interest.
