# Supplementary material for: Comparison of Multiple Methods for Determination of FCGR3A/B Genomic Copy Numbers in HapMap Asian Populations with Two Public Databases
Source: Front Genet. 2016 Dec 26;7:220. doi: 10.3389/fgene.2016.00220 (PMC5183586; doi:10.3389/fgene.2016.00220)
Supplement: Supplementary file 1 [file Table1.DOCX]

**An improved comparison of multiple methods for determination of *FCGR3A/B* genomic copy numbers in HapMap Asian populations**

Yuan-yuan Qi^1.2.3.4¶^, Xu-jie Zhou^1.2.3.4¶^, Ding-fang Bu^5^, Ping Hou^1.2.3.4^, Ji-cheng Lv^1.2.3.4^, Hong Zhang^1.2.3.4^*

**AUTHORS’ INSTITUTION AND AFFILIATION**

1. Renal Division, Peking University First Hospital,

2. Peking University Institute of Nephrology,

3. Key Laboratory of Renal Disease, Ministry of Health of China,

4. Key Laboratory of Chronic Kidney Disease Prevention and Treatment (Peking University), Ministry of Education, Beijing, People’s Republic of China

5. Research Central Institute, Peking University First Hospital, Beijing, People’s Republic of China

*** CORRESPONDING AUTHOR**

Hong Zhang, MD, PhD

Renal Division, Peking University First Hospital,

Peking University Institute of Nephrology,

Key Laboratory of Renal Disease, Ministry of Health of China,

Key Laboratory of Chronic Kidney Disease Prevention and Treatment (Peking University), Ministry of Education, Beijing, People’s Republic of China

No. 8, Xishiku Street, Xicheng District

Beijing 100034, P.R China

Tel.: +86 10 83572388;

Fax: +86 10 66551055.

Email: [hongzh@bjmu.edu.cn](mailto:hongzh@bjmu.edu.cn)

¶ Yuan-yuan Qi and Xu-jie Zhou contributed equally to this work.

| **S1 Table. FCGR3 copy numbers in CHB and JPT populations by TaqMan, PRT-REDVR, SYBR Green and STR.** | | | | | | | | | | | |
| --- | --- | --- | --- | --- | --- | --- | --- | --- | --- | --- | --- |
| **Sample** | **Population** | **TaqMan** | |  | **PRT-REDVR** | |  |  | **SYBR Green** |  | **STR** |
|  |  | **Copy Numbers** | **AB** |  | **Copy Numbers** | **AB** | **HNA1a1b** |  | **B** |  | **Copy Numbers** |
| NA18524 | CHB | 4 | AABB |  | 3 | AAB | 1a |  | BB |  | 4 |
| NA18526 | CHB | 5 | AABBB |  | 6 | AAABBB | 1a1b1b |  | BBB |  | 5 |
| NA18529 | CHB | 5 | AABBB |  | 6 | AABBBB | 1a1a1b1b |  | BBB |  | 5 |
| NA18532 | CHB | 4 | AABB |  | 4 | AABB | 1a1b |  | BBB |  | 4 |
| NA18537 | CHB | 5 | AAABB |  | 4 | AABB | 1b1b |  | BBB |  | 5 |
| NA18540 | CHB | 4 | AABB |  | 4 | AABB | 1a1b |  | BB |  | 4 |
| NA18542 | CHB | 4 | AABB |  | 6 | AAABBB | 1a1b1b |  | BB |  | 4 |
| NA18545 | CHB | 5 | AAABB |  | 5 | AAABB | 1b1b |  | BBB |  | 5 |
| NA18547 | CHB | 4 | AABB |  | 3 | AAB | 1a |  | B |  | 3 |
| NA18550 | CHB | 4 | AABB |  | 4 | AABB | 1b1b |  | BB |  | 4 |
| NA18552 | CHB | 5 | AABBB |  | 5 | AABBB | 1a1a1b |  | BBB |  | 6 |
| NA18555 | CHB | 6 | AABBBB |  | 5 | AABBB | 1a1a1b |  | BBBB |  | 6 |
| NA18558 | CHB | 5 | AAABB |  | 5 | AAABB | 1a1a |  | BBB |  | 6 |
| NA18561 | CHB | 4 | AABB |  | 4 | AABB | 1a1b |  | BBB |  | 4 |
| NA18562 | CHB | 4 | AABB |  | 4 | AABB | 1a1a |  | BB |  | 4 |
| NA18563 | CHB | 4 | AABB |  | 4 | AABB | 1a1b |  | BB |  | 4 |
| NA18564 | CHB | 2 | AB |  | 2 | AB | 1a |  | B |  | 2 |
| NA18566 | CHB | 4 | AABB |  | 3 | AAB | 1a |  | BB |  | 4 |
| NA18570 | CHB | 4 | AABB |  | 4 | AABB | 1a1a |  | BBBB |  | 4 |
| NA18571 | CHB | 5 | AABBB |  | 5 | AABBB | 1a1a1b |  | BBB |  | 6 |
| NA18572 | CHB | 4 | AABB |  | 4 | AABB | 1a1b |  | BB |  | 4 |
| NA18573 | CHB | 3 | AAB |  | 3 | AAB | 1b |  | BB |  | 3 |
| NA18576 | CHB | 4 | AABB |  | 4 | AABB | 1a1b |  | BB |  | 4 |
| NA18577 | CHB | 4 | AABB |  | 4 | AABB | 1b1b |  | BBB |  | 5 |
| NA18579 | CHB | 5 | AABBB |  | 6 | AAABBB | 1a1b1b |  | BBB |  | 5 |
| NA18582 | CHB | 3 | AAB |  | 3 | AAB | 1a |  | B |  | 3 |
| NA18592 | CHB | 6 | AAABBB |  | 6 | AAABBB | 1a1a1b |  | BBB |  | 4 |
| NA18593 | CHB | 4 | AABB |  | 4 | AABB | 1b1b |  | BB |  | 4 |
| NA18594 | CHB | 5 | AABBB |  | 5 | AABBB | 1a1a1b |  | BBB |  | 5 |
| NA18603 | CHB | 7 | AAABBBB |  | 7 | AAABBBB | 1a1a1b1b |  | BBBB |  | 6 |
| NA18605 | CHB | 5 | AABBB |  | 5 | AABBB | 1a1a1b |  | BBB |  | 5 |
| NA18608 | CHB | 4 | AABB |  | 4 | AABB | 1a1a |  | BB |  | 6 |
| NA18609 | CHB | 4 | AABB |  | 4 | AABB | 1a1a |  | BB |  | 4 |
| NA18611 | CHB | 4 | AABB |  | 4 | AABB | 1a1b |  | BB |  | 4 |
| NA18612 | CHB | 5 | AABBB |  | 5 | AABBB | 1a1a1b |  | BBB |  | 5 |
| NA18620 | CHB | 4 | AABB |  | 4 | AABB | 1a1b |  | BB |  | 4 |
| NA18621 | CHB | 5 | AABBB |  | 5 | AABBB | 1a1b1b |  | BBB |  | 5 |
| NA18622 | CHB | 4 | AABB |  | 4 | AABB | 1a1b |  | BB |  | 4 |
| NA18623 | CHB | 4 | AABB |  | 4 | AABB | 1a1b |  | BB |  | 4 |
| NA18624 | CHB | 4 | AABB |  | 4 | AABB | 1b1b |  | BB |  | 4 |
| NA18632 | CHB | 4 | AABB |  | 5 | AABBB | 1a1a1b |  | BB |  | 4 |
| NA18633 | CHB | 5 | AABBB |  | 5 | AABBB | 1a1b1b |  | BBB |  | 5 |
| NA18635 | CHB | 4 | AABB |  | 4 | AABB | 1b1b |  | BB |  | 5 |
| NA18636 | CHB | 5 | AAABB |  | 5 | AAABB | 1a1a |  | BBB |  | 4 |
| NA18637 | CHB | 4 | AABB |  | 4 | AABB | 1a1b |  | BBB |  | 4 |
| NA18940 | JPT | 3 | AAB |  | 3 | AAB | 1a |  | BB |  | 3 |
| NA18942 | JPT | 4 | AABB |  | 4 | AABB | 1a1b |  | BB |  | 4 |
| NA18943 | JPT | 4 | AABB |  | 4 | AABB | 1a1b |  | BB |  | 4 |
| NA18944 | JPT | 4 | AABB |  | 4 | AABB | 1a1b |  | BB |  | 4 |
| NA18945 | JPT | 4 | AABB |  | 4 | AABB | 1a1a |  | BB |  | 4 |
| NA18947 | JPT | 4 | AABB |  | 4 | AABB | 1a1a |  | BB |  | 4 |
| NA18948 | JPT | 4 | AABB |  | 4 | AABB | 1a1a |  | BB |  | 4 |
| NA18949 | JPT | 3 | AAB |  | 3 | AAB | 1a |  | BB |  | 4 |
| NA18951 | JPT | 4 | AABB |  | 4 | AABB | 1a1b |  | BB |  | 5 |
| NA18952 | JPT | 5 | AABBB |  | 5 | AABBB | 1a1b1b |  | BBB |  | 5 |
| NA18953 | JPT | 4 | AABB |  | 4 | AABB | 1a1a |  | BB |  | 4 |
| NA18956 | JPT | 5 | AABBB |  | 6 | AABBBB | 1a1a1b1b |  | BBB |  | 5 |
| NA18959 | JPT | 4 | AABB |  | 4 | AABB | 1a1b |  | BB |  | 4 |
| NA18960 | JPT | 5 | AABBB |  | 5 | AABBB | 1a1a1b |  | BBB |  | 5 |
| NA18961 | JPT | 4 | AABB |  | 4 | AABB | 1a1a |  | BB |  | 4 |
| NA18964 | JPT | 4 | AABB |  | 4 | AABB | 1a1a |  | BB |  | 4 |
| NA18965 | JPT | 4 | AABB |  | 5 | AAABB | 1a1a |  | BB |  | 4 |
| NA18966 | JPT | 4 | AABB |  | 4 | AABB | 1a1a |  | BB |  | 4 |
| NA18967 | JPT | 5 | AABBB |  | 5 | AABBB | 1a1a1b |  | BBB |  | 5 |
| NA18968 | JPT | 4 | AABB |  | 4 | AABB | 1a1a |  | BB |  | 4 |
| NA18969 | JPT | 4 | AABB |  | 4 | AABB | 1a1a |  | BB |  | 4 |
| NA18970 | JPT | 3 | AAB |  | 3 | AAB | 1a |  | BB |  | 4 |
| NA18971 | JPT | 6 | AAABBB |  | 6 | AAABBB | 1a1b1b |  | BBB |  | 5 |
| NA18972 | JPT | 4 | AABB |  | 5 | AABBB | 1a1a1b |  | BB |  | 4 |
| NA18973 | JPT | 4 | AABB |  | 4 | AABB | 1a1a |  | BB |  | 3 |
| NA18974 | JPT | 5 | AABBB |  | 5 | AABBB | 1a1b1b |  | BBB |  | 5 |
| NA18975 | JPT | 6 | AAABBB |  | 4 | AABB | 1a1a |  | BB |  | 4 |
| NA18976 | JPT | 5 | AABBB |  | 4 | AABB | 1a1b |  | BB |  | 4 |
| NA18978 | JPT | 4 | AABB |  | 4 | AABB | 1b1b |  | BBB |  | 4 |
| NA18980 | JPT | 4 | AABB |  | 4 | AABB | 1b1b |  | BB |  | 4 |
| NA18981 | JPT | 6 | AAABBB |  | 6 | AAABBB | 1a1a1a |  | BB |  | 4 |
| NA18987 | JPT | 5 | AAABB |  | 5 | AAABB | 1a1a |  | BBBB |  | 5 |
| NA18990 | JPT | 4 | AABB |  | 4 | AABB | 1a1a |  | BB |  | 4 |
| NA18991 | JPT | 4 | AABB |  | 5 | AABBB | 1a1b1b |  | BB |  | 4 |
| NA18992 | JPT | 4 | AABB |  | 4 | AABB | 1b1b |  | BBB |  | 4 |
| NA18994 | JPT | 4 | AABB |  | 4 | AABB | 1a1b |  | BB |  | 4 |
| NA18995 | JPT | 3 | AAB |  | 4 | AAAB | 1a |  | BB |  | 3 |
| NA18997 | JPT | 4 | AABB |  | 3 | AAB | 1a |  | BB |  | 4 |
| NA18998 | JPT | 6 | AAABBB |  | 6 | AAABBB | 1a1a1a |  | BBBB |  | 7 |
| NA18999 | JPT | 5 | AABBB |  | 6 | AABBBB | 1a1a1b1b |  | BBB |  | 5 |
| NA19000 | JPT | 4 | AABB |  | 4 | AABB | 1a1a |  | BB |  | 4 |
| NA19003 | JPT | 5 | AABBB |  | 6 | AAABBB | 1b1b1b |  | BBB |  | 5 |
| NA19005 | JPT | 4 | AABB |  | 4 | AABB | 1a1b |  | BB |  | 4 |
| NA19007 | JPT | 4 | AABB |  | 4 | AABB | 1a1b |  | BB |  | 4 |
| NA19012 | JPT | 4 | AABB |  | 4 | AABB | 1a1a |  | BBB |  | 4 |
